# Supplementary figures and images for: LncRNAs Are Differentially Expressed between Wildtype and Cell Line Strains of African Trypanosomes
Source: Noncoding RNA. 2022 Jan 12;8(1):7. doi: 10.3390/ncrna8010007 (PMC8788480; doi:10.3390/ncrna8010007)

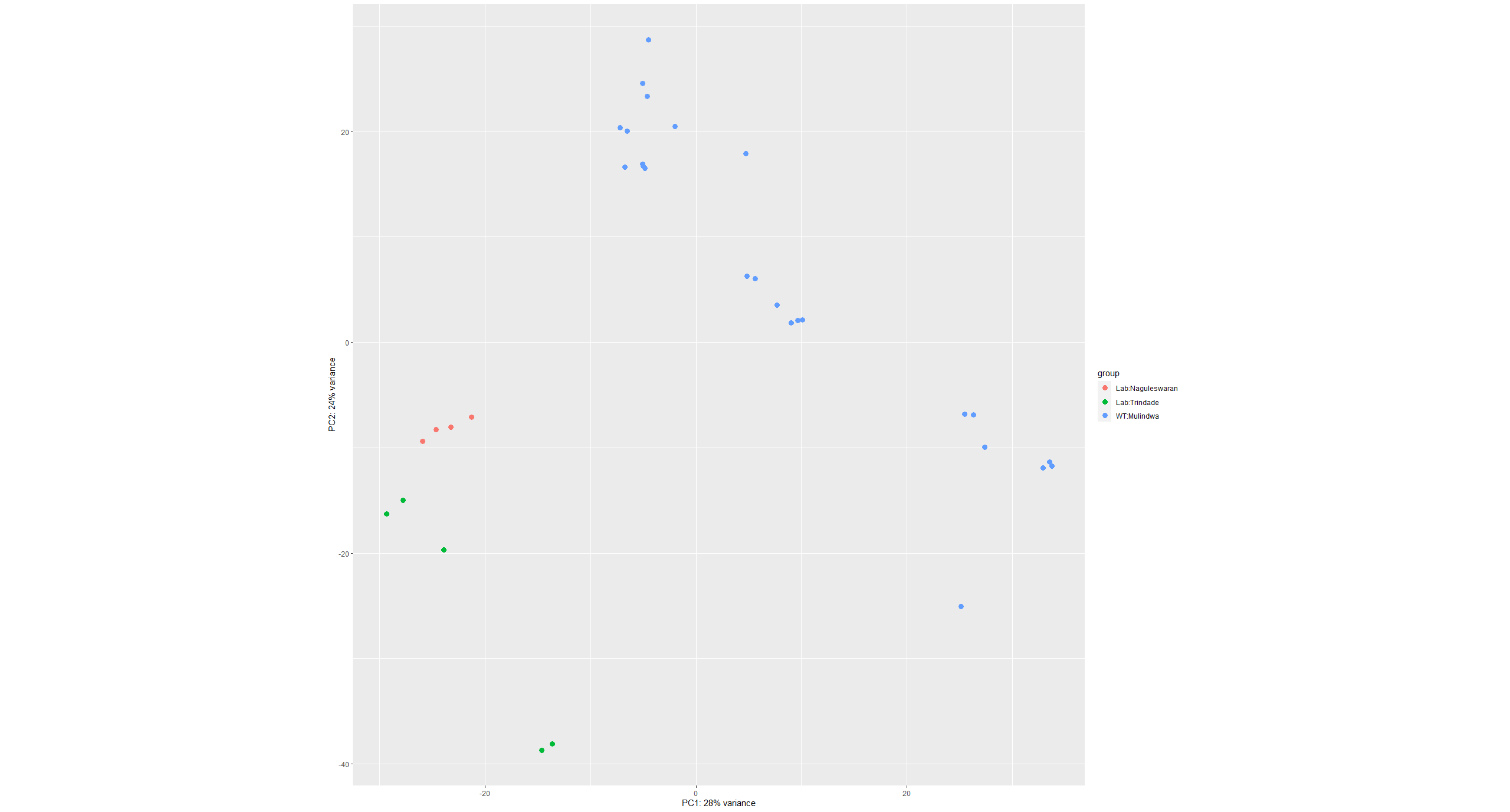

Supplement: Supplementary file 1 [file ncrna-08-00007-s001.zip › Sup_figure1_PCA_plot.tif]

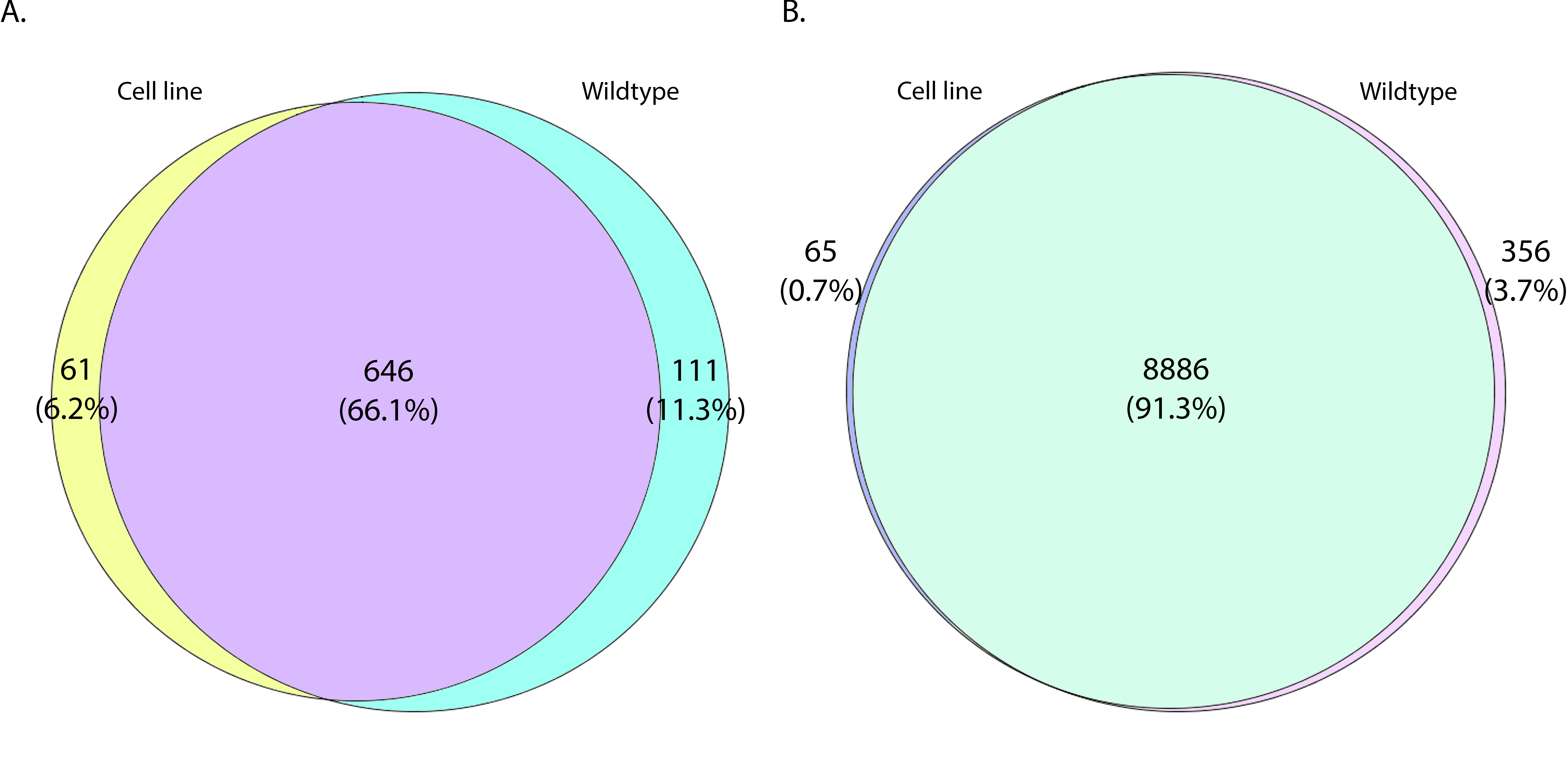

Supplement: Supplementary file 1 [file ncrna-08-00007-s001.zip › Sup_figure2_venn_nc1.tif]

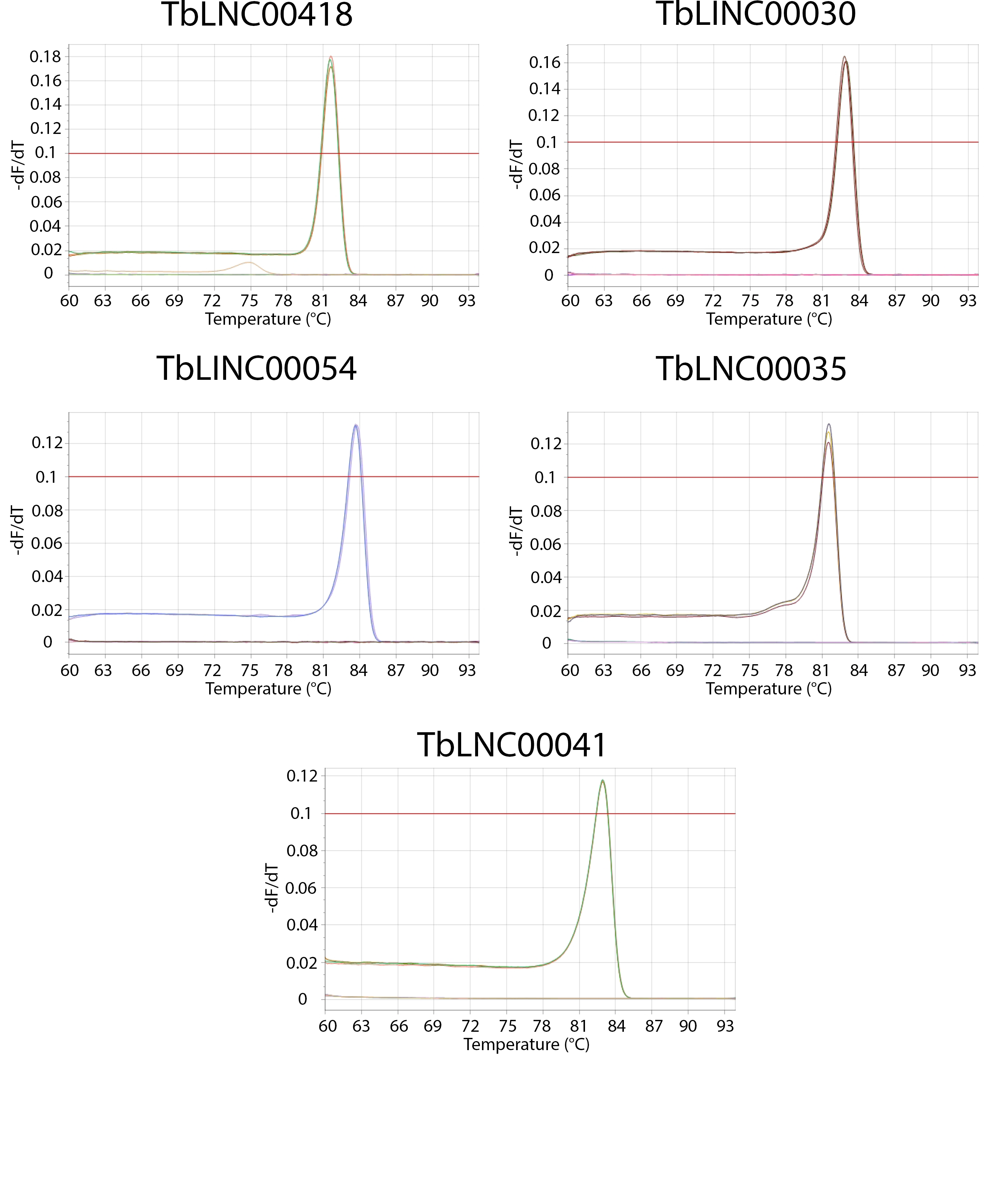

Supplement: Supplementary file 1 [file ncrna-08-00007-s001.zip › Sup_figure3_melt_curves.tif]
